# Modelagem da Política Nacional de Promoção da Saúde e as sinuosidades da sua implementação

**DOI:** 10.1590/0102-311XPT133025

**Published:** 2026-02-06

**Authors:** Maria Socorro de Araújo Dias, Maria da Conceição Coelho Brito, Lielma Carla Chagas da Silva, Dais Gonçalves Rocha, Anya Pimentel Gomes Fernandes Vieira-Meyer

**Affiliations:** 1 Universidade Estadual Vale do Acaraú, Sobral, Brasil.; 2 Universidade de Brasília, Brasília, Brasil.; 3 Fiocruz Ceará, Eusébio, Brasil.

**Keywords:** Promoção da Saúde, Política Pública, Avaliação em Saúde, Health Promotion, Public Policy, Health Evaluation, Promoción de la Salud, Política Pública, Evaluación en Salud

## Abstract

Buscou-se analisar a modelagem teórico-lógica da implementação da Política Nacional de Promoção da Saúde (PNPS). Para isso, realizou-se um Estudo de Avaliabilidade (EA). A coleta ocorreu em 2022 e 2023. O cenário envolveu nove estados, o Distrito Federal e 48 municípios. Foram acessados 291 documentos e 276 informantes-chave. Elaborou-se um Modelo Teórico-Lógico (MTL), um dos produtos do EA, tendo este sido validado em cinco oficinas regionais com 112 participantes. Pela complexidade, a PNPS requer a constituição de um MTL com representação imagiológica distinta de MTL convencionais, que possuem sequência unidirecional. Com o MTL, foi possível clarificar Princípios e Valores, Diretrizes, Componentes, Atividades e Recursos envolvidos na implementação da PNPS nas diversas regiões do país. Os resultados são discutidos em quatro seções: o problema e as confluências que estruturam a PNPS (problema e contexto); inspirações e direcionalidades na implementação da PNPS (princípios, valores e diretrizes); subsídios e movimentos que concretizam a PNPS (recursos físicos, organizacionais e simbólicos); e desenlaces e potências da PNPS: entre o que é e o vir a ser (atividades; ações, resultados e monitoramento e avaliação). Os resultados evidenciam a institucionalização da PNPS por políticas locais e ações voltadas às doenças crônicas não transmissíveis. No Monitoramento e Avaliação (M&A), não foram identificados indicadores específicos, o que incide na necessidade de investimentos no M&A da PNPS. Não há indicadores de impacto, contudo, reconhece-se impacto nas interações entre os elementos que conformam o design da implementação da PNPS com potencial de respostas ao problema original. Há articulação da PNPS com outras políticas.

## Abstract (en)

This study sought to analyze a theoretical-logical model of the implementation of the Brazilian National Policy of Health Promotion (PNPS, acronym in Portuguese) via an Evaluability Assessment (EA). Data were collected from 2022 to 2023. The scenario involved nine states, the Federal District, and 48 municipalities. In total, 291 documents and 276 key informants were searched. A Theoretical-Logical Model (TLM, an EA product) was elaborated and validated in five regional workshops with 112 participants. Due to its complexity, the PNPS requires a TLM with an imaging representation unlike that of conventional TLM (which have a unidirectional sequence). The chosen TLM evinced the principles, values, guidelines, components, activities, and resources in the implementation of the PNPS across Brazil. The discussion of the results has four sections: the problem and the confluences that structure the PNPS (problem and context); inspirations and directionalities in implementing the PNPS (principles, values, and guidelines), subsidies and movements that materialized the PNPS (physical, organizational, and symbolic resources), and the PNPS outcomes and strengths: between what is and what comes to be (activities, actions, results, monitoring, and evaluation). The results show the institutionalization of the PNPS by local policies and actions aimed at chronic noncommunicable diseases. Monitoring and Evaluation showed no specific indicators, evincing the need to invest in them within the PNPS. Despite no indicators, the impact of the interactions between the elements that make up the PNPS implementation design may meet the needs of the original problem. The PNPS includes articulation with other policies.

## Abstract (es)

Se buscó analizar la modelación teórico-lógica de la implementación de la Política Nacional de Promoción de la Salud (PNPS) de Brasil. Para ello, se realizó un Estudio de Evaluabilidad (EE). La recopilación de datos se llevó a cabo en 2022 y 2023. El escenario abarcó nueve estados, el Distrito Federal y 48 municipios. Se consultaron 291 documentos y 276 informantes clave. Se elaboró un Modelo Teórico-Lógico (MTL), uno de los productos del EE, que fue validado en cinco talleres regionales con 112 participantes. Debido a su complejidad, la PNPS requiere la constitución de un MTL con una representación imagística distinta de los MTL convencionales, que tienen una secuencia unidireccional. Con el MTL, fue posible aclarar los principios y valores, las directrices, los componentes, las actividades y los recursos involucrados en la implementación de la PNPS en las diversas regiones del país. Los resultados se discuten en cuatro sesiones: el problema y las confluencias que estructuran la PNPS (problema y contexto); inspiraciones y orientaciones en la implementación de la PNPS (principios, valores y directrices); subsidios y movimientos que concretan la PNPS (recursos físicos, organizativos y simbólicos); y desenlaces y potencialidades de la PNPS: entre lo que es y lo que será (actividades; acciones, resultados y seguimiento, y evaluación). Los resultados evidencian la institucionalización de la PNPS mediante políticas locales y acciones orientadas a las enfermedades crónicas no transmisibles. En el Monitoreo y la Evaluación (M&E), no se identificaron indicadores específicos, lo que incide en la necesidad de inversiones en el M&E de la PNPS. No hay indicadores de impacto, sin embargo, se reconoce el impacto en las interacciones entre los elementos que conforman el diseño de la implementación de la PNPS con potencial para dar respuesta al problema original. Existe articulación de la PNPS con otras políticas.

## Introdução

A Saúde Coletiva, por se expressar como um espaço social que concentra abordagens relacionadas à concretização do direito à saúde ^1^, denota sinergia com a Promoção da Saúde (PS) - campos de construções sociais comprometidos com os modos de vida e possibilidades de bem viver de pessoas e coletivos ^2^.

A Política Nacional de Promoção da Saúde (PNPS), instituída em 2006 e redefinida em 2014, assume o objetivo de promover a equidade e a melhoria das condições e modos de vida da população, reduzindo vulnerabilidades e riscos decorrentes de processos de determinações sociais, econômicas, políticas, culturais e ambientais. Assim, propõe a interlocução de saberes científicos, técnicos e populares, bem como a mobilização de recursos institucionais, setoriais, comunitários públicos e privados a favor da qualidade de vida ^3^ ^,^ ^4^.

Reconhece-se que a necessária tangibilidade e os compromissos anunciados por uma política pública demandam investidas analíticas ^5^ como atividade essencial à promoção da transparência sobre sua implementação e subsídios para tomada de decisão. Logo, a proposição de Estudo de Avaliabilidade (EA) da PNPS reveste-se de importância, especialmente quando se considera que esta política se ocupa do enfrentamento de problemas complexos ^6^ ^,^ ^7^. Ademais, muitas avaliações de políticas públicas no Brasil desconsideram uma matriz de valores e privilegiam a eficiência e/ou a conformidade administrativa, não incorporando a complexidade envolvida nos desenhos institucionais e operacionais, tornando-as um projeto isolado de coordenação centralizada ^5^.

Este estudo, portanto, endossa uma perspectiva plural consonante à complexidade do campo da PS, considerando os interessados na implementação, o desenho político e os arranjos legais e normativos ^5^ da PNPS. Assim, objetiva-se analisar a modelagem teórico-lógica da implementação da PNPS, como elemento base de uma pré-avaliação.

## Método

O EA representa um processo avaliativo realizado em alguma fase do desenvolvimento e da implementação de uma política. Diante disso, apoia a definição de ações dirigidas à transformação de contextos no campo da Saúde Coletiva, visando considerar interlocuções realistas de uma política pública, sua viabilidade, aceitabilidade e adaptação, melhorando seu desempenho na garantia de direitos sociais ^8^. Sua coleta de dados ocorreu entre março de 2022 e setembro de 2023, sustentada em análise documental e escutas a informantes-chave da implementação da PNPS ^8^ ^,^ ^9^.

De abrangência nacional, a seleção dos estados participantes considerou: ser estado respondente ao levantamento de práticas de PS realizadas entre maio e setembro de 2021, pelo Departamento de Prevenção e Promoção da Saúde (DEPROS) do Ministério da Saúde; a representação de cada região brasileira, de modo a expressar diversidade de cenários; e diferentes Índices de Desenvolvimento Humano (IDH) e porte populacional. Isso resultou na representação: Nordeste (Ceará, Maranhão e Pernambuco); Norte (Tocantins); Centro-oeste (Goiás e Distrito Federal); Sudeste (Minas Gerais e São Paulo); e Sul (Rio Grande do Sul e Paraná). Participaram, portanto, nove estados e o Distrito Federal.

**Figura 1:**
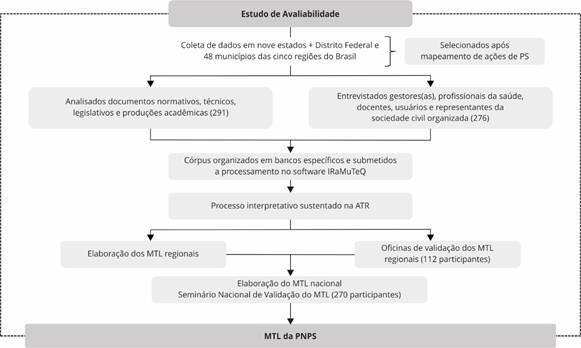
Diagrama de detalhamento do método. Brasil, 2022-2023.

ATR: Análise Temática Reflexiva; DF: Distrito Federal; MTL: Modelo Teórico-Lógico; PS: Promoção da Saúde. Fonte: elaboração própria.

Aos municípios pertencentes aos estados selecionados ocorreu a aplicação de um questionário de mapeamento de PS pela equipe de pesquisadoras. Aos municípios respondentes (n = 61), aplicaram-se os mesmos critérios para definição dos estados, assegurando cinco municípios/regiões administrativas por estado/Distrito Federal, um de cada porte populacional. Dos 50 municípios selecionados, 48 aceitaram integrar essa pesquisa (Figura 1).

Foram acessados 998 documentos, sendo 291 selecionados e submetidos à análise. Esta adotou um instrumento elaborado pelas autoras para extração de informações relativas ao problema que originou a ação, a descrição da ação, os princípios, os objetivos, a população/público-alvo, os recursos necessários à implementação, os produtos, os resultados e os fatores e/ou contextos de influência.

Informantes-chave foram identificados a partir da técnica *snowball* (bola de neve) ^10^, um processo de recrutamento em que os participantes iniciais foram gestores (um vinculado à Secretaria de Saúde do Estado e outro vinculado à Secretaria de Saúde do Município), ambos envolvidos com a implementação da PNPS. A partir desses participantes, outros foram incluídos mediante uma cadeia de indicação para reconhecer personagens implicados com a implementação da PNPS nos cenários.

Assim, participaram gestores(as), profissionais da saúde, docentes, usuários e representantes da sociedade civil organizada, totalizando 276 interessados. Nas entrevistas, adotaram-se dois roteiros semiestruturados (um para gestores, profissionais e docentes e outro para usuários e sociedade civil), elaborados pelas autoras e submetidos a pré-teste. Os instrumentos contemplavam: caracterização dos participantes, entendimentos e concepções de PS, ações desenvolvidas, estratégias de monitoramento e avaliação, alcances da PNPS, participação da comunidade, avanços e desafios. O tempo total das entrevistas foi de 160 horas, 11 minutos e 20 segundos.

Os dados foram sistematizados em planilhas no Microsoft Excel (https://products.office.com/). Após, houve a análise textual pelo IRaMuTeQ, versão 0.7 (http://www.iramuteq.org/). O processo interpretativo e de base para a elaboração do Modelo Teórico-Lógico (MTL) da PNPS sustentou-se na Análise Temática Reflexiva (ATR), técnica que estuda um fenômeno pela interpretação e pelo contexto em que os dados são produzidos ^11^.

Como um dos produtos do EA, foi elaborado o MTL da PNPS, estrutura conceitual e operacional que subsidia a tomada de decisão ^8^ ^,^ ^9^. Esclarece-se que esse artigo advém da *Pesquisa de Avaliabilidade da Política Nacional de Promoção da Saúde*. Essa objetivou compreender o desenho de implementação da PNPS desenvolvida em diferentes territórios brasileiros, a partir de um MTL, e propor perguntas avaliativas e as recomendações para melhores práticas. O MTL é um produto sobre o qual se dedica esse artigo.

A base imagiológica do MTL da PNPS contemplou os elementos: Problema, Contexto externo indutor, Contexto político regional, Princípios e valores, Diretrizes, Componentes, Recursos, Atividades, Resultados, Monitoramento e avaliação, Conexão com outras políticas ou programas e Impacto.

A construção do MTL da PNPS deu-se a partir da elaboração de MTLs regionais, que foram sistematizados e, posteriormente, validados. A estruturação e validação do MTL da PNPS reconheceram a necessidade de sinergia com os territórios; assim, foram conduzidas cinco oficinas (uma por região), que contaram 112 dos 276 participantes da fase de coleta de dados, assegurando representação de estados e municípios. Esclarece-se que as oficinas foram orientadas pela Técnica do Grupo Nominal (TGN) para geração de consensos ^12^. As discussões, em cada oficina, foram sistematizadas em uma matriz de consistência e subsidiaram alinhamentos nos MTLs regionais e, assim, no nacional. Após as oficinas regionais, ocorreu um Seminário Nacional, no qual o MTL da PNPS foi validado; este contou com 270 participantes, com representação do DEPROS, Organização Pan-Americana da Saúde (OPAS), gestores e trabalhadores estaduais e municipais da PS, pesquisadores da área e sociedade civil.

Pesquisa aprovada em distintos Comitês de Ética em Pesquisa: da Universidade Estadual Vale do Acaraú (pareceres nº 5.132.031, nº 5.317.460 e nº 5.542.218); da Secretaria Municipal da Saúde de Belo Horizonte (parecer nº 6.127.987); da Fundação de Ensino e Pesquisa em Ciências da Saúde da Secretaria de Saúde do Distrito Federal (parecer nº 5.586.062); do Hospital do Trabalhador da Secretaria de Saúde do Paraná (parecer nº 5.593.264); e da Secretaria Municipal da Saúde de Curitiba (parecer nº 6.096.552).

## Resultados e discussão

Os resultados que estruturam a expressividade e traduções da PNPS nos diversos contextos advêm de diversas vozes de atores envolvidos em sua implementação. O perfil de participantes assumido como necessário para a compreensão da PNPS converge, portanto, com a multiplicidade de escopos de ação para sua concretização. Na Tabela 1, apresenta-se a caracterização dos 276 participantes desta pesquisa.

**Tabela 1:** Distribuição dos informantes-chave por perfil e cenário da pesquisa. Brasil, 2022-2023.

Região/UF	Gestor(a)	Profissional de saúde	Docente	Usuário(a)	Total
n (%)	n (%)	n (%)	n (%)	n (%)
Nordeste					
Ceará	13 (56,5)	6 (26,1)	1 (4,3)	3 (13,0)	23 (8,3)
Maranhão	22 (73,3)	6 (20,0)	-	2 (6,7)	30 (10,9)
Pernambuco	27 (75,0)	7 (19,4)	-	2 (5,6)	36 (13,0)
Norte					
Tocantins	11 (57,9)	5 (26,3)	1 (5,3)	2 (10,5)	19 (6,9)
Centro-oeste					
Distrito Federal	15 (57,7)	4 (15,4)	2 (7,7)	5 (19,2)	26 (9,4)
Goiás	10 (50,0)	6 (30,0)	4 (20,0)		20 (7,2)
Sudeste					
Minas Gerais	13 (50,0)	9 (34,6)	3 (11,5)	1 (3,8)	26 (9,4)
São Paulo	21 (51,2)	13 (31,7)	7 (17,1)	-	41 (14,9)
Sul					
Paraná	8 (32,0)	14 (56,0)	3 (12,0)	-	25 (9,1)
Rio Grande do Sul	5 (16,7)	17 (56,7)	3 (10,0)	5 (16,7)	30 (10,9)

Fonte: dados da pesquisa.

Ao reconhecer que, além do operativo, importa o contexto regulamentador, foram analisados 291 documentos nas cinco regiões brasileiras (Tabela 2). Destes, 131 (45%) eram do tipo normativo, 75 (25,8%) de natureza técnica, 65 (22,3%) legislativa e 12 (4,1%) de produção acadêmica.

**Tabela 2:** Distribuição dos documentos analisados por região. Brasil, 2022-2023.

Região	Documentos	
	n	%
Nordeste	81	27,8
Norte	15	5,2
Centro-oeste	35	12,0
Sul	98	33,7
Sudeste	62	21,3

Fonte: dados da pesquisa.

A integração e interpretação dos corpus analíticos subsidiaram a elaboração do MTL da PNPS. A PNPS, ao propor enfrentamentos a um problema complexo, requer uma representação imagiológica distinta de MTL convencionais, de sequência unicausal ou unidirecional ^13^ ^,^ ^14^. Essa representação gráfica irradiada de valores e intencionalidades está disponível no repositório institucional da Fundação Oswaldo Cruz (ARCA; https://arca.fiocruz.br/items/a7fa8b2f-27d8-4bba-83ba-4a1aeed92301).

Os elementos constitutivos do MTL da PNPS foram interpretados e organizados em unidades temáticas que evidenciam aproximações necessárias, suas interações e alcances, conforme se anuncia: o problema e as confluências que estruturam a PNPS (problema e contexto); inspirações e direcionalidades que se introjetam na implementação da PNPS (princípios, valores e diretrizes); subsídios e movimentos (e seus paradoxos) que concretizam a PNPS (recursos físicos, organizacionais e simbólicos); e desenlaces e potências da PNPS - entre o que é e o vir a ser (atividades, ações, resultados, monitoramento, avaliação e impacto).

### O problema e as confluências que estruturam a PNPS

No MLT, circunscreve-se toda a conformação da PNPS a partir de um problema originário que influencia e é influenciado por debates em torno da PS. Assim, o problema se refere a “*iniquidades e o fazer predominantemente fragmentado, individualizante, dissociado do ambiente e de base curativista*”, a partir do qual se reafirma o compromisso assumido pela PNPS ^2^ ao apresentar o objetivo de “*promover a equidade e a melhoria das condições e dos modos de viver, ampliando a potencialidade da saúde individual e coletiva e reduzindo vulnerabilidades e riscos à saúde decorrentes dos determinantes sociais, econômicos, políticos, culturais e ambientais*”.

O problema da PNPS expressa uma defesa da reversão de um sistema provocador de abismos sociais que condicionam diferenças sistemáticas e relevantes, mas também evitáveis, injustas e desnecessárias ^15^. Assim, investir em práticas de cuidado integradoras, intersetoriais e sistêmicas é estratégia de promoção da equidade em saúde, tida como capaz de suplantar contextos desfavoráveis ao pleno da pessoa.

Há uma urgência ainda latente, mas reconhecível, de que a abordagem às iniquidades em saúde tem como essência a priorização da equidade em políticas públicas, pois melhoram resultados de saúde tidos como fulcrais ao enfrentamento de gradientes sociais injustos ^16^. Isso endossa o papel social insurgente da PNPS ao propor movimentos favoráveis à consecução da equidade.

Nesse ínterim, retoma-se à necessária interlocução da PNPS com contextos indutores, expressos em marcos e fatos históricos, sociais e políticos. Assumiu-se no MTL, como Contexto externo, os escopos internacionais e nacionais. Na dimensão internacional, delimitam-se eventos em temporalidade abrangente (entre 1975 e 2016), denotando debate permanente sobre práticas de cuidado efetivas e equânimes. Nesse interstício, a Conferência de Alma Ata (1978) demarca a promulgação do direito aos cuidados essenciais da saúde e da atenção primária à saúde (APS), convergindo como movimento mundial em defesa da saúde como direito e dever de todos ^17^.

Por sua vez, o Contexto externo internacional refletiu no nacional. A APS ganhou notoriedade no Brasil com a criação do Programa Saúde da Família (PSF) e tornou-se um espaço potente da PS ^18^. As práticas desta se tornaram tão sinérgicas à APS que, hoje, tem na sua secretaria um departamento de PS, além desta estar circunscrita no cuidado em saúde de diretriz operacional da Política Nacional de Atenção Básica (PNAB).

Destacaram-se, também, no Contexto internacional, a Conferência de Ottawa (1986) e as 8ª e 9ª Conferências Mundiais de Promoção da Saúde com os temas *Saúde em Todas as Políticas* e *Promoção da Saúde nos Desenvolvimentos Sustentáveis*, respectivamente realizadas em 2013 e 2016. As temáticas reafirmaram a intenção de tornar a PS um espaço político e orientador de práticas sociais equânimes para enfrentamento de determinações que afetam as pessoas em seus cotidianos.

É evidente o escopo de ações voltadas às doenças crônicas não transmissíveis (DCNT), pauta que foi proposta pela Estratégia Global em Alimentação Saudável, Atividade Física e Saúde (2003) e que impulsionou agenda global e nacional expressa, inclusive, no Plano de Ações Estratégicas para o Enfrentamento das Doenças Crônicas e Agravos Não Transmissíveis no Brasil (2021-2030). Vinculados a este Plano, foram referidos no contexto externo nacional o Programa Academia da Saúde (PAS) e o Programa Nacional de Controle do Tabagismo (PNCT).

Outro movimento internacional foi a Agenda 2030 (Objetivos do Desenvolvimento Sustentável - ODS). Especialmente nas regiões Centro-oeste e Sul, os ODSs foram referidos nas estratégias e/ou ações, assim como nos indicadores para a PS. Nesta perspectiva, estão sendo desencadeadas ações intersetoriais envolvendo meio ambiente, a exemplo das experiências Programa Câmbio Verde e Curitiba 2035, em Curitiba (Paraná). Também dialoga com a agenda o movimento de cidades e municípios saudáveis com liderança da OPAS no Brasil. Estas experiências coadunam com a premissa de que a intersetorialidade amplia o cuidado sob a égide da integralidade, responsabilização e resolutividade ^19^.

No âmbito nacional, destacam-se as políticas promotoras de equidade identificadas em três regiões (Norte, Sudeste e Sul), que apresentam interfaces com o objetivo da PNPS. Isto refletiu-se na indução de ações de PS com populações em situações de vulnerabilidades (p.ex.: população quilombola, ribeirinhas e refugiados).

A partir do Contexto externo indutor, os movimentos referidos também são reconhecidos como “marcos nacionais e internacionais” motivadores da revisão da PNPS de 2014 ^2^ ^,^ ^9^. Isso porque permitiram lançar olhares sensíveis para um debate contundente para operacionalização da PS no cenário nacional e mediado por sua própria PNPS, oportunizando maior institucionalidade da discussão.

A PNPS conta, ainda, com um Contexto político interno constituído por políticas e mecanismos de implementação. Das dez Unidades Federativas (UF) participantes, cinco têm políticas estaduais ou distrital (Distrito Federal, Minas Gerais, Goiás, Paraná e Ceará) e dois políticas em suas capitais (Goiânia e Curitiba). Já considerando dispositivos de implementação, sinalizam-se comitês setoriais e intersetoriais: câmara intersecretarial; núcleos de prevenção à violência e promoção da saúde; e redes, como a rede de municípios e cidades saudáveis referidas em Goiás e Pernambuco, e a Rede de Centros Urbanos de Cultura, Arte, Ciência e Esporte (Rede CUCA), no Ceará.

Fóruns e observatórios destacaram-se como estratégias de troca de experiências e mapeamento de práticas de PS. Tais espaços fomentam o *advocacy* pela PS e podem apoiar movimentos de suplantação de práticas individuais e biomédicas ^20^. Sob o mesmo escopo, os conselhos de saúde, *locus* de defesa e de pactuação da agenda da PS, atuam como promotores de garantias necessárias ao bem-viver. As políticas de PS dos estados e municípios participantes desta pesquisa foram aprovadas nesta instância.

Os Programas Federais de Segurança Viária e Família Cidadã foram citados como possibilitadores de ações de PS intersetoriais. A Lei anti-fumo emergiu como estratégia legislativa e ambiental para consecução do tema da PNPS “*enfrentamento ao uso de tabaco e seus derivados*”. Ainda, foram mencionados o Núcleo Ampliado de Saúde da Família (NASF) e Programas de Residências Multiprofissionais, por favorecerem a integralidade da atenção ^21^.

UFs do Nordeste, Centro-oeste, Sul e Sudeste têm implementado programas de Promoção da Igualdade Racial e Políticas de Equidade que dialogam com valores relativos à diversidade expressos na PNPS ^2^. Essas iniciativas são necessárias, especialmente quando se considera haver uma relação entre racismo, discriminação e resultados em saúde ^22^.

Na interlocução da diversidade e do equânime acesso à saúde, respeitando as singularidades e os modos de viver, retoma-se a imperativa confluência entre desejos, expectativas e resultados da relação entre universidades, serviços e comunidade; logo, o encontro desses cenários em ideário, prática e consciência coletiva pode proporcionar uma efetiva práxis promotora de saúde. Logo, universidades, instituições e núcleos de pesquisa são destacados no MTL, o que valoriza o compromisso de fortalecer, apoiar e avaliar a consecução das políticas públicas, colaborando com uma visão ampliada de PS e conhecimento da PNPS.

As análises evidenciaram que o Contexto político interno exerce um papel impulsionador da PNPS, em uma translação da influência, também, do Contexto externo indutor. Há, contudo, que se retomar ao Problema que motivou a PNPS e que influencia suas engrenagens.

### Inspirações e direcionalidades que se introjetam na implementação da PNPS

A base principiológica e valorativa da PNPS é anunciada como Princípios e Valores que intentam maior coesão entre a operacionalização e o problema, pois atuam como um gradiente que visa minimizar a desvirtualização das engrenagens e perda do sentido dos movimentos: defesa do bem-estar como um direito singular, equânime e compromisso coletivo.

Os Princípios e Valores da PNPS são indutores das ações nos territórios, aspecto que justifica suas expressões, ainda que em intensidades diferentes, nos discursos dos informantes-chave e documentos analisados. É oportuno referir o reconhecimento de princípios que norteiam outras políticas nacionais e que guardam importante articulação com a PNPS, a saber: Amorosidade (Política Nacional de Educação Popular em Saúde), Transversalidade (Política Nacional de Humanização) e Universalidade (princípio doutrinário do Sistema Único de Saúde - SUS). Outrossim, emergiram, também, os princípios Igualdade, Compromisso e Resiliência em algumas regiões; isso reafirma a singularidade dos contextos de implementação da PNPS.

As Diretrizes, por sua vez, assumem papel de orientação da PNPS nos territórios. Há destaques necessários: cooperação e articulação intra e intersetorial; planejamento de ações territorializadas; gestão democrática, participativa e transparente; ampliação da governança; e estímulo à pesquisa. Reflete-se que tais Diretrizes, além de serem previstas pela Política, sobressaem-se nos cenários participantes por interconectarem as práticas de PS a partir de um compromisso comum assumido por diversos setores da sociedade, que respeitam a construção social da pessoa no território, com práticas de gestão responsáveis e sustentáveis em prol de um poder social e compartilhado ^2^.

Houve, ainda, singularidades nas políticas locais de PS. Elas acrescem a ampliação da capacidade institucional para o desenvolvimento efetivo das práticas de PS, e a defesa das práticas integrativas e complementares em saúde (PICS) (demarcada na Política Estadual do Ceará), o que denota a preocupação com a institucionalidade das ações, além da valorização de um cuidado que soma saberes do território; esse aspecto ainda é endossado pela incorporação da Educação Popular como diretriz (nas Políticas Estaduais do Ceará e de Minas Gerais).

Coadunante, a comunicação foi referenciada como diretriz (Política Municipal de Promoção da Saúde de Curitiba), o que denota sua essencialidade para uma PS sensível aos modos de viver das pessoas e suas diversidades. Ademais, houve diretrizes sobre o estímulo à comunicação, responsabilidade pela articulação intersetorial e previsão de orçamento (Política Municipal de Promoção da Saúde de Goiânia).

As singularidades do MTL reafirmam a premissa de que uma política pública deve considerar necessidades em saúde locorregionais, estabelecendo uma captura destas e transversalizando-as em uma implementação horizontal da PNPS. Assim, no MTL da PNPS são apresentados Componentes que convergem com os temas transversais e os eixos operacionais da PNPS; sobre este elemento do MTL, é oportuno enfatizar a formação e educação permanente em diálogo com a epistemologia da Educação Popular em Saúde (EPS) para subsidiar as práticas de PS.

Há sintonia entre a EPS e a PS, pois desvela um olhar permeado de aprendizados e que considera contextos críticos que ensejam uma ação mais específica. Desse modo, a dimensão analítica inerente a EPS, em âmbito social, cultural, econômico e político, favorece o desenvolvimento mais efetivo de processos de mudança em situações e relações promotoras de exclusão ^23^. Essa perspectiva transformadora é uma defesa emancipatória da PNPS.

A efetivação da PNPS como práxis transformadora do Problema, que considera contextos e direcionalidades, requer a disponibilização de Recursos de distintas naturezas para o desenvolvimento de Atividades intrínsecas ou correlatas.

### Subsídios e movimentos (e seus paradoxos) que concretizam a PNPS

A implementação da PNPS requer acessar, além dos convencionais recursos físicos e organizacionais, os simbólicos. Pois, concebe-se que o pensar, o sentir e o agir humano tornam-se fundamentais para o envolvimento de gestores, trabalhadores e sociedade para implementação da PS ^24^.

Na análise dos Recursos disponíveis, destaca-se a importância atribuída ao financiamento federal, reconhecendo a responsabilidade tripartite. Também foi referida, com ênfase, a institucionalização da PS na estrutura organizacional das secretarias estaduais e/ou municipais da saúde.

Como recursos simbólicos reconhecidos, tidos como arranjos organizacionais informacionais e atitudinais, valores e conceitos, destacam-se: afetividade; ancestralidade; saberes populares; comunicação, escuta e motivação; reconhecimento de experiências, interprofissionalidade e trabalho em equipe; sensibilização dos gestores e vontade política; pertencimento e conhecimento do território; e conexão com os ODS. Ressalta-se que a interprofissionalidade e o trabalho em equipe ativam a agenda de PS nos territórios, o que fortalece o entendimento da integralidade do cuidado ^25^. Entretanto, foi destacada, no curso da coleta de dados, a descontinuidade do NASF-Atenção Básica (NASF-AB) e do incentivo financeiro como fatores críticos à implementação da PS. Registra-se a atual iniciativa de reestabelecimento das equipes multiprofissionais ampliadas por meio do e-Multi ^26^.

As Atividades mencionadas como evidências de implementação da PNPS demonstram que são sustentadas, predominantemente, em uma agenda programática. Emergiram, também, ações de educação em saúde e de prevenção a doenças e agravos, estando esta última vinculada a programas federais. Reflete-se que a prática de PS traduzida, essencialmente, em agendas de programas nacionais, a faz perder ou minimizar o seu caráter territorializado e inovador como resposta às necessidades locais.

Ao assumir o território como produtor das necessidades de cuidado, é oportuno debater o papel da comunidade nas práticas da PS. Contudo, no MTL da PNPS, é expresso que o protagonismo de pessoas e lideranças, e ações de empoderamento da população, no eixo participação e controle social, apresenta menor expressividade de implementação, aspecto realçado na dificuldade da participação de representantes da população nesta pesquisa. As vozes da população a quem se destina a política pública devem ser asseguradas na avaliação desta, introjetando o vivencial à perspectiva teórica; isso se sustenta na intencionalidade necessária de superação das narrativas dominantes que desconsideram marcadores sociais da vida das pessoas ^27^.

Argumenta-se, ainda, que a mudança necessária ao enfrentamento dos determinantes em saúde enseja a incorporação político-pedagógica da pessoa e dos coletivos. Pois, o empoderamento promove a abertura de espaços necessários para a transformação do cotidiano e privilegia uma coconstrução do cuidado em saúde em uma perspectiva, sobretudo, social ^28^.

### Desenlaces e potências da PNPS: entre o que é e o vir a ser

Ao investir na análise dos Resultados apresentados no MTL, destacam-se a institucionalização da PNPS por meio de políticas locais e a difusão desta. Outros resultados indicam o crescimento das ações e redução dos indicadores das DCNT com ênfase na promoção da alimentação adequada e saudável. Informa-se que as DCNT, além de terem constituído agenda prioritária da PNPS em sua primeira versão, tem sido pautado o seu enfrentamento nos planos estaduais e municipais.

A ênfase relativa às DCNT é tida como global, visto que estas são responsáveis por 41 milhões de mortes de pessoas a cada ano, o equivalente a 74% de todas as mortes no mundo. Ademais, a oportunidade de elencá-las entre as atividades da PNPS é necessária, pois, a cada ano, 17 milhões de pessoas morrem de alguma DCNT antes dos 70 anos, sendo que 86% dessas mortes ocorrem em países de baixa e média renda; tais dados reafirmam a necessidade de uma intervenção abrangente ^29^, integrada e intersetorial, e que seja capaz de agir nos determinantes sociais que imprimem escolhas não tão individuais assim.

Identifica-se, também, número expressivo e institucionalização de ações de educação, prevenção e informação (Setembro Amarelo, Outubro Rosa e Novembro Azul). Estas atividades de natureza pontual e sazonal são relatadas como oportunidades de PS, vinculando esta à noção de prevenção. Esta compreensão anuncia desafios para um processo de implementação sólida da PNPS.

Os movimentos que debatem a PS percorrem uma história com avanços de significados e práticas, mas ainda há críticas relacionadas ao predomínio de ações voltadas à prevenção e recuperação da saúde. Logo, reflete-se sobre os distanciamentos na prática do cuidado em saúde, especialmente ao considerar a PNPS; na consecução das atividades de uma política não somente a teoria e o objetivo interferem, mas, também, as formas como os serviços se organizam, quais as concepções de saúde e de cuidado para os profissionais envolvidos, além das possíveis disputas de interesses e questões políticas ^30^.

Ao sumarizar os Resultados do MTL, reconhece-se consonância com o tema prioritário *Promoção da Mobilidade Segura*, conforme se destaca: promoção da mobilidade segura, ambientes saudáveis e sustentáveis; ampliação do projeto Vida no Trânsito; e redução da taxa de mortalidade por acidentes de trânsito. Similarmente, quanto ao tema *Promoção da Cultura de Paz e Direitos Humanos*, reconhece-se a implementação da atenção psicossocial nos territórios. Em tempo, evidenciam que as ações de prevenção da violência assumem uma visibilidade importante e necessária ^31^.

A PS se ocupa de múltiplos aspectos da vida humana, em uma defesa pela justiça como compromisso social e de respeito ao outro. A PNPS, ao considerar a saúde como uma concepção ampliada, reconhece que o ambiente seja seguro, saudável e sustentável, promovendo adequada mobilidade humana e qualidade de vida, mediante integração entre os setores da sociedade.

No Monitoramento & Avaliação (M&A), há destaque para ações voltadas a esse âmbito a partir dos instrumentos de gestão e dos percentuais de acompanhamento e cobertura das condicionalidades do Programa Bolsa Família. Ainda foram considerados como indicadores de M&A o número de produções técnicas (boletins, eventos, fóruns, publicações e materiais produzidos) e de parcerias com institutições de Ensino Superior (IES). Foram, também, relacionados a indicadores do Sistema de Informação em Saúde para a Atenção Básica (os relacionados a atividades coletivas) e a programas específicos (Programa Saúde na Escola, Projeto Vida no Trânsito, PNCT, Academia da Saúde e de DCNT).

A implementação de políticas públicas enseja acompanhamento das suas ações. Quando se trata da PS, investir em M&A reveste-se de complexidade perante as nuances que atravessam sua operacionalização. Pois, a PNPS se dedica a fomentar (re)construções sociais e de sujeitos sob premissa emancipatória, logo, diversa. Nesse entendimento, o anunciado no MTL guarda tímida observância analítica da implementação das ações de PS, uma vez que oferece poucas pistas sobre os atributos verificáveis e os impactos inerentes a estes. Esta situação é corroborada por Fontes et al. ^32^ (p. 3) quando afirmam serem “*escassas pesquisas que relacionem este tema à gestão, especificamente aos instrumentos de planejamento*”. Isso culmina em desafios urgentes relacionados ao M&A da PNPS.

Outrossim, os indicadores presentes no MTL permitem refletir que as escolhas podem estar associadas a tradições e expertises próprias das instituições para encontrar em seus acervos alternativas para operacionalização de indicadores oficiais. Assim, podem existir diferenças entre os indicadores oficiais e aqueles efetivamente operacionalizados ^33^.

Investir em processos coerentes de M&A das políticas públicas favorece a consecução do compromisso social inerente a elas. Isso porque correspondem à definição de planos e coordenações, movimentos de formação, vigilância e busca por informações que fomentem tomada de decisão e aprendizagens coerentes ao impacto esperado da política, conferindo maiores possibilidades de sustentabilidade desta ^34^.

A PNPS prevê articulação com outras políticas públicas para fortalecê-la, especialmente por reafirmar a impossibilidade do setor saúde atender, de forma isolada, ao enfrentamento dos determinantes da saúde ^2^. Consonante, o MTL inclui o elemento Conexão com outras políticas e programas, no qual se revela uma diversidade de movimentos com outras políticas, programas e estratégias para o enfrentamento do problema ocupado pela PNPS.

No desenvolvimento de políticas públicas, há expectativa de impactos favoráveis à sociedade, visto serem concebidas como iniciativas voltadas à transformação de uma realidade social percebida como problemática ^7^. Ao retomar o MTL da PNPS, e por incursões reflexivas já realizadas no decorrer desse artigo, não foi possível dimensionar um impacto verificável em métricas, até porque não há, ainda, indicadores específicos que traduzam a complexidade da PNPS. Contudo, sob uma perspectiva abrangente, a PNPS já transforma, apesar de avanços necessários, os territórios em que é implementada, pois a interação entre as forças constituintes (elementos do MTL) age de forma real e com potência de resposta ao Problema que transcende relação de causalidade, mas que pode amplificar seu alcance para outras dimensões que superam as esperadas.

Diante dos resultados apresentados, considera-se que a análise da modelagem teórico-lógica da implementação da PNPS permitiu: (1) apreensão dos marcos e singularidades indutores da PNPS; (2) convergência dos valores, princípios e diretrizes com o marco de referência da PNPS; (3) a inclusão de recursos simbólicos, evidenciando mobilização necessária para a efetivação da PS; e (4) que as ações programáticas ainda prevalecem em detrimento daquelas com princípios de participação social e empoderamento. Desta maneira, no MTL, os resultados da PNPS também refletiram a agenda programática, sendo que houve maior visibilidade em alguns estados para os temas prioritários *Promoção da Mobilidade Segura* e *Promoção da Cultura de Paz e Direitos Humanos*.

## Conclusão

Este estudo analisou a modelagem teórico-lógica da PNPS, demonstrando que, apesar dos avanços na implementação da PNPS, ainda existem desafios significativos, como a falta de indicadores para mensurar seu impacto e a necessidade de articulação intersetorial.

Os resultados obtidos têm implicações diretas para gestores e formuladores de políticas, que devem priorizar a formação de profissionais e o desenvolvimento de indicadores robustos para a avaliação da PNPS. Contudo, é necessário destacar que, dada a complexidade do problema que originou a PNPS e a heterogeneidade dos territórios estudados, reconhece-se impacto nas interações entre os elementos que conformam seu design de implementação com potencial de respostas ao problema original, embora não se expresse em uma relação de linearidade. Logo, entende-se que seus efeitos transcendem métricas esperadas e ampliam o alcance de sua implementação.

Essa pesquisa fornece investimentos necessários à sustentabilidade da PNPS. À medida que o contexto social e de saúde do Brasil se transforma, é essencial que a PNPS se adapte para responder de forma eficaz às novas necessidades, garantindo que a promoção da saúde se torne uma prioridade em todas as esferas da sociedade. As evidências traduzem diversas realidades brasileiras, aspecto que endossa a confiabilidade e validade desta Pesquisa; entretanto, utilizaram-se representações (criteriosamente definidas) para acessar essas realidades, o que implica em cautela na tradução e translação destes resultados para outros contextos.

Outrossim, os resultados despertam para outros escopos de análise. Diante disso, recomenda-se a implementação de processos de educação permanente com os profissionais de saúde e a criação de um sistema de monitoramento e avaliação que permita acompanhar a implementação da PNPS de forma efetiva.

## Data Availability

Os dados de pesquisa estão disponíveis mediante solicitação à autora de correspondência.
